# Timing of shoot development transitions affects degree of perenniality in *Arabidopsis lyrata* (Brassicaceae)

**DOI:** 10.1186/s12870-015-0606-2

**Published:** 2015-09-17

**Authors:** David L. Remington, Jennifer Figueroa, Mitali Rane

**Affiliations:** Department of Biology, University of North Carolina at Greensboro, P.O. Box 26170, Greensboro, NC 27402 USA

## Abstract

**Background:**

Perenniality is best understood in quantitative terms, involving the relationship between production vs. turnover of meristems, biomass, or energy reserves. Previous quantitative trait locus (QTL) studies using divergent populations of the perennial rock cress *Arabidopsis lyrata* have shown that trade-offs in vegetative growth vs. reproduction are due to cascading effects of differences in early vegetative development, which contribute to local adaptation. However, details of the developmental differences and how they affect perenniality remained unclear. In this study, we investigated in detail the developmental differences in perenniality between populations. *A. lyrata* from Norway and North Carolina populations, representing contrasting environments and degrees of perenniality, were grown under controlled conditions, and data were collected on plant phenology and shoot-level development. We tested hypotheses that differences in perenniality involve strict allocation of lateral meristems to vegetative vs. reproductive fates, or alternatively quantitative effects of pre-reproductive vegetative development.

**Results:**

The two populations showed large differences in the degree of vegetative development on individual shoots prior to reproductive transitions. The number of leaves produced on shoots prior to bolting, and not strict meristem allocation or variation in apical dominance, was able to explain variation in the number of inflorescences on individual plants. These results suggested that allocation of time to shoot vegetative vs. reproductive development could be a major factor in resource allocation differences between the populations.

**Conclusions:**

Based on these results and those of previous QTL studies, we propose a model in which the degree of shoot vegetative development shapes the developmental context for reproduction and subsequent vegetative growth in different environments. Climate-specific effects of shoot development patterns on reproductive output and survival may result in divergent evolutionary trajectories along a perenniality continuum, which may have broader relevance for plant life history evolution.

## Background

Land plants have evolved a spectacular range of variation in life histories. At one extreme are trees that can live for hundreds or even thousands of years, prompting the question of whether perennial plants truly undergo aging [1–3]. At the other are semelparous plants, mostly annuals and biennials but including some monocarpic perennials, which die after a single bout of reproduction. While death in semelparous annuals may seem programmed at first glance, the differences between annuals and perennials are probably best understood in quantitative terms [4]. Perenniality can be characterized by the persistence of indeterminate vegetative meristems and in many cases green leaf tissue through the entire reproductive season and beyond [1, 4]. Some species, such as *Mimulus guttatus* and *Erysimum capitatum* include both annual and perennial genotypes, which differ in the numbers of vegetative and reproductive shoots they produce [5–8]. *Sorghum bicolor*, an annual crop grass that can be made perennial by cultural practices, harbors substantial genetic variation for leaf senescence, with several mapped quantitative trait loci (QTL) affecting the timing or rate of leaf senescence [9]. Thus, differences in rates of production vs. turnover of meristems, tissue, or energy reserves are key factors governing where species or particular genotypes lie on a perenniality continuum [3].

From this perspective, perenniality is closely associated with greater resource allocation to growth and somatic maintenance at the expense of current reproduction. Resource allocation is typically described in terms of limited energetic resources allocated to alternative processes [10–12], but in plants it can also be modeled in terms of alternative meristem fates [13]. Meristem allocation models are based on the distinction that vegetative and inactive meristems can remain indeterminate, but commitment of meristems to reproduction is with few exceptions irreversible, leading to consumption of the meristem by the end of the reproductive season [4, 13]. Apical dominance can have a key role in governing meristem fates and thus perenniality, but there is conflicting evidence on its relationship to life history. A comparison of congeneric pairs of semelparous and iteroparous plant species found that iteroparity was associated with stronger apical dominance, presumably because suppressed axillary meristems remain available for future vegetative growth [14]. However, detailed comparisons of annual vs. perennial genotypes in *Erysimum capitatum* [8] and *Mimulus guttatus* [5] indicate that iteroparity is favored by greater lateral branching prior to reproduction. In these latter cases, lateral branches persist as vegetative shoots, leading to perenniality. Iteroparity in wild-type *Arabis alpina* vs. precociously-flowering mutants is also associated with the extent to which lateral shoots formed after vernalization remain vegetative through the reproductive season [15, 16]

The perennial rock cress species *Arabidopsis lyrata* (L.) O’Kane and Al-Shehbaz is a promising experimental system for deciphering the relationship between genetic, developmental and evolutionary processes shaping the perenniality continuum. *A. lyrata* has a wide but patchy circumpolar distribution, and grows primarily in low-competition environments ranging from subarctic to warm temperate in climate [17–19]. Populations from different locations show moderate to high levels of molecular differentiation [18, 19] and strong differentiation in fitness-related traits [20–24]. *A. lyrata* belongs to a perennial sister lineage to the well-characterized annual *A. thaliana* [17, 25], and has a published complete genome sequence and well-established synteny to *A. thaliana* that facilitate identification of genes with adaptive significance [26–28].

Previous reciprocal-transplant studies have shown distinct contrasts among *A. lyrata* populations in reproductive investment and life history, with populations from cold (Spiterstulen, Norway) and warm (Mayodan, North Carolina USA) environments exemplifying constrasting degrees of perenniality. Natural populations at both sites are clearly perennial, though differences in the frequency of plants with spreading, highly branched vegetative mats suggest that average longevity is greater at Spiterstulen. When plants representing several unrelated families from each population were grown together, Spiterstulen plants showed lower propensity to flower and produced fewer inflorescences than Mayodan plants in both North Carolina and Norway environments [21]. Mayodan plants showed much lower year-to-year survival than Spiterstulen plants when grown in Norway, while both populations showed poor survival after the first reproductive season in North Carolina, indicating environment-dependent differences between populations in perenniality. These factors contributed to fitness advantages for each population in their local environments [21].

Quantitative trait locus (QTL) analyses using outcross F_2_ progeny of crosses between these populations planted at the same two study sites found that a combination of conditionally neutral and antagonistically pleiotropic QTL regions contributed to the fitness advantage of the local populations [29]. Strong trade-offs between reproduction and vegetative growth differentiated the two populations when grown in North Carolina but not in Norway [30]. The much higher reproductive output of the Mayodan plants in North Carolina was accompanied by major reductions in vegetative diameter during the reproductive period, while Spiterstulen plants increased their vegetative diameter on average during this period. The trade-offs in North Carolina resulted from the coordinated effects of several QTL regions on vegetative growth patterns and multiple components of reproductive output. Mayodan alleles at some of these same QTL regions reduced survival in Norway but did not increase reproductive output, indicating that QTL effects on survival were not due to direct costs of reproduction. Structural equation modeling of QTL effects indicated that cascading effects of QTL on early vegetative growth patterns generated the coordinated effects on resource allocation in North Carolina. The results provided indirect evidence that Spiterstulen plants have weaker apical dominance than Mayodan plants, with more lateral vegetative branch development prior to the start of reproduction precluding subsequent reproductive growth. QTL effects were shifted to later in development in Norway, explaining the absence of coordinated effects of QTL on resource allocation there.

**Fig. 1:**
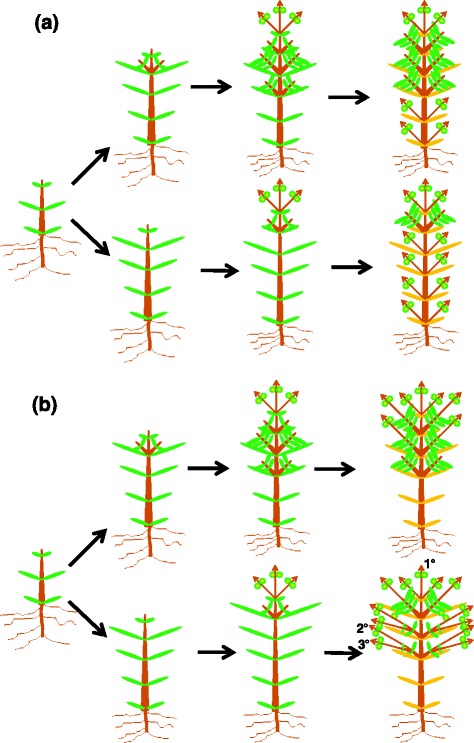
Diagram showing alternative hypotheses to explain contrasting life history patterns in *A. lyrata*. **a** Strict meristem allocation hypothesis, in which inflorescences develop only from meristems that did not start vegetative development before the onset of reproduction. Genotypes that produce more vegetative shoots before starting reproduction (top row) would thus produce fewer inflorescences than those with fewer vegetative shoots (bottom row). **b** Quantitative inhibition hypothesis, in which plants with weaker apical dominance (top row) undergo more extensive vegetative development from lateral shoots prior to reproduction, which then produce fewer inflorescences than plants that maintain stronger apical dominance prior to reproduction (bottom row). Examples of first-order (1°), second-order (2°), and third-order (3°) shoots are shown on the bottom right panel. Internodes within the vegetative crown (all portions of plant except inflorescences) are elongated to illustrate branching pattern

Those findings [30] provide evidence that the Spiterstulen and Mayodan *A. lyrata* populations occupy strongly contrasting positions on a perenniality continuum, but provide only limited information on the developmental mechanisms that are involved. Here, we report a more detailed study of vegetative and reproductive development in these divergent populations, conducted under controlled conditions that allowed us to characterize the developmental basis for the contrasting life history patterns. One hypothesis is that a strict meristem allocation process occurs, in which lateral meristems that develop vegetatively before the onset of flowering remain vegetative through the ensuing reproductive season (Fig. 1a). Under this hypothesis, inflorescences would develop only from meristems that remained dormant prior to the onset of reproduction, which are more abundant on Mayodan plants. Thus, we would predict that inflorescence-bearing shoots would show little or no evidence of vegetative development prior to bolting. An alternative hypothesis is that pre-reproductive vegetative development of axillary meristems inhibits subsequent production of inflorescences quantitatively rather than categorically (Fig. 1b). If so, differences among individual plants in measures of apical dominance (i.e. repression of lateral vegetative shoot development before flowering) would largely account for the between-population difference in number of inflorescences, with Mayodan plants showing greater apical dominance. We grew plants under conditions that simulated a long growing season in order to evaluate the full developmental trajectory of each population over a single cycle of growth and reproduction. However, the results provide insights on mechanisms that could explain the contrasting effects on relative fitness seen under short growing seasons in Norway. We discuss the broader relevance of our results to the evolution of perenniality.

## Methods

### Study organism

The development pattern in *A. lyrata* is similar in many respects to that described in *A. thaliana* [31]. The primary shoot develops as a compact vegetative rosette, which gives rise to a terminal inflorescence upon transition of the shoot apical meristem to a reproductive fate. Axillary meristems give rise to lateral shoots which can form additional inflorescences. Detailed observations in *A. lyrata* (D.L. Remington, unpublished data) indicate that axillary meristems are typically activated in a basipetal (apical to basal) progression either before or after the reproductive transition of the shoot apical meristem. As in *A. thaliana*, the more basal cauline nodes within the inflorescence often produce elongated branches rather than individual flowers, resulting in branched inflorescences. In some genotypes, multiple orders of inflorescence branching may occur. Unlike *A. thaliana*, most populations of *A. lyrata* are self-incompatible and have somewhat larger, more showy white flowers that are pollinated by insects. Carpels of pollinated flowers mature into elongated modified capsules (siliques) with 10-40 seeds.

In contrast with *A. thaliana*, some lateral shoots in *A. lyrata* undergo extensive vegetative development, sometimes resulting in many unelongated lateral vegetative shoots branching from within the primary rosette. In addition, at least some *A. lyrata* genotypes produce shoots from short rhizomes, which generally emerge near the primary shoot and may contribute to survival [18]. Our observations of plants grown under controlled conditions have suggested that plants from different populations differ both in their propensity to produce lateral vegetative shoots and to produce rhizomatous shoots. Lateral vegetative shoots commonly produce smaller leaves than the primary shoot, leading to the observation that plants with extensive vegetative branching tend to have smaller vegetative diameters than plants with unbranched rosettes [30]. Perenniality in *A. lyrata* is a consequence of lateral vegetative shoots or rhizomatous shoots that persist beyond the reproductive season without undergoing a reproductive transition.

### Plant materials

*A. lyrata* seed originating from populations from Spiterstulen, Norway (61° 38´N, 8° 24´E,1106 m.a.s.l.) and Mayodan, North Carolina USA (36°25′ N, 79°58′ W, 225 m.a.s.l.) were used in this study. Seed from Spiterstulen were obtained from Outi Savolainen (University of Oulu, Finland), and consisted of four unrelated full-sib families from crosses between plants grown from field-collected seed. Seed from Mayodan were collected in the field in 2010, and consisted of open-pollinated maternal families.

### Growing conditions

**Fig. 2:**
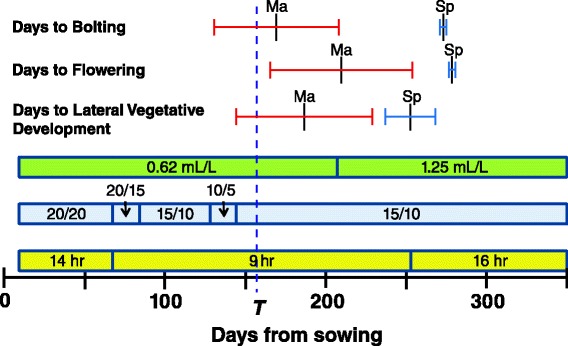
Timeline showing photoperiod lengths (yellow bar), day/night temperatures (°C; blue bar), and fertilizer concentrations (green bar) used over the course of the study. The date of transplanting (*T*) is indicated with a dashed blue line. The mean (±2 s.d.) days to first bolting, days to first flowering, and days to first lateral shoot development are shown for Mayodan (Ma) and Spiterstulen (Sp) plants, excluding plants that did not bolt or flower over the course of the study

Seeds from four Spiterstulen full-sib families and six Mayodan half-sib families were sown in Fafard Germinating mix in 125 cm^3^ plastic cells, with 60 cells per plastic flat. A total of 18 seeds were sown per family, six seeds in each of 3 flats. Flats were covered with plastic lids and placed in the dark at 4 °C for nine days, then transferred to a growth chamber with 14 hr/10 hr light/dark cycles at 20 °C, approximating late summer conditions under which *A. lyrata* seedlings typically germinate in North Carolina. Subsequently, the photoperiod and temperature conditions were adjusted periodically to approximate the progression of fall, winter, and spring conditions in North Carolina (Fig. 2). After most of the germinated seedlings had two true leaves, plastic lids were removed, and plants were watered 3x/week and fertilized bi-weekly with a solution of 0.62 mL L^-1^ 24-8-16 fertilizer with micronutrients (Miracle-Gro). At 157 days post-germination, plants with their intact germinating mix plugs were transferred to plastic cups 7.6 cm diameter × 15 cm deep filled with a fritted clay media (Turface All Sport). The germinating mix-fritted clay combination was intended to mimic typical *A. lyrata* growing environments in North Carolina, in which plants are typically found growing in patches of organic litter and duff occurring in rock outcrops. Plants were placed in portable racks by population (4 or 5 plants/rack) and these were placed so that the populations were distributed around the growth chamber. Locations of plants within the growth chamber were regularly rotated. At 207 days post-germination, the fertilizer concentration was increased to 1.25 mL L^-1^ for bi-weekly fertilization.

### Data collection

Seed germination was recorded by cell, identified by family, a consecutive seed number (1-18), and the flat in which it was located. Germinated seedlings were only obtained from two of the four Spiterstulen families. For each germinating seedling, the date of first visible lateral vegetative bud development, the date of first bolting (i.e. visibly elongated inflorescence shoot), and the date of first flowering were recorded. At the time of first bolting, the vegetative diameter of each plant was measured as the longest distance between vegetative (non-cauline) leaf tips. At that time, the degree of lateral vegetative shoot development (branchiness) was also rated on a 1-5 scale as a measure of apical dominance, with 1 representing no visible lateral buds, and 5 representing a rosette structure dominated entirely by lateral shoots (Table 1). The number of emerged inflorescences was recorded at intervals throughout the reproductive period.

**Table 1:** Rating system for apical dominance (branchiness)

Rating	Description
1	All visible rosette leaves are primary leaves (on main stem, not emerging from lateral shoots). All newer leaves (not fully elongated yet) are attached above the older, fully-elongated leaves. Primary shoot apex is obvious and dominant, and the leaves extend horizontally from it.
2	Some leaves emerging from lateral shoots are visible but are much smaller than fully-elongated primary leaves. Some newer leaves are obviously attached below larger leaves on main stem. Primary shoot apex is obvious and still clearly dominant over lateral vegetative shoots.
3	Leaves from lateral shoots are apparent, and some may be difficult to distinguish from primary leaves. The primary shoot apex is still apparent but is losing its dominance, and some lateral shoots are nearly as vigorous as the main shoot. The vegetative crown is beginning to acquire a bushy form, with many leaves in a vertical orientation.
4	Many lateral shoot leaves are nearly as large as the primary leaves. The primary and lateral shoot apices are becoming difficult to distinguish, though larger primary leaves produced earlier may still be apparent on the lower part of the plant. The vegetative has a bushy form, with leaves extending at all angles.
5	The primary and lateral shoots can no longer be distinguished. All fully-elongated leaves are relatively compact. The vegetative crown has a dense cushiony appearance, with leaves extending at all angles.

Between 329 and 350 days after sowing, when development of new inflorescences had begun to taper off, plants from a representative subset of racks from each population were selected for detailed morphological analyses (27 Mayodan plants and 10 Spiterstulen plants). On each of these plants, the total number of inflorescences was counted, and all inflorescence-bearing shoots were carefully removed. Branch order for each inflorescence-bearing shoot was determined by careful visual inspection, with the main shoot being 1^st^-order, lateral shoots emerging directly from the main shoot being 2^nd^-order, lateral shoots emerging from 2^nd^-order shoots being 3^rd^-order, and so forth (Fig. 1b, bottom right panel). Each instance of bolting from an apical or axillary position on the unbolted portion of a shoot was recorded as a separate inflorescence. Inflorescence shoots emerging from cauline leaves produced above the position of bolting were not considered to be separate inflorescences. For each 2^nd^-order and higher-order inflorescence, the number of basal vegetative leaves produced before reproductive transition (i.e. bolting) was counted or estimated, and the length of the largest basal leaf was recorded. It was assumed that each *n* + 1-order shoot emerging from the basal, non-bolted portion of an *n*th-order shoot had been subtended by a leaf, so the number of basal leaves recorded for an *n*th-order shoot was always at least the number of *n* + 1-order shoots even if no basal leaves were visible. It is possible that some of the shoots recorded as *n* + 1-order were actually *n*th-order shoots that developed from accessory buds, as it was difficult to determine the point of origin for some shoots. All shoots emerging from rhizomes were recorded as 2^nd^-order.

### Statistical analysis

All statistical analyses were done using R version 3.0.2 [32]. Effects of population on pre-reproductive vegetative diameter were tested for all plants in the study with linear models using the lm function in R, and were log transformed to improve homoscedasticity. Days to bolting, flowering, and first visible lateral vegetative shoot development had highly skewed and heteroscedastic distributions, and some plants did not demonstrate these traits prior to dying or reaching the end of the experiment. Thus, effects of population on these traits were tested with Cox proportional hazards models, which incorporate such right-censored data, using the coxph function in the R survival package. Mean number of vegetative leaves per shoot, mean length of the largest basal leaf on each shoot, the number of inflorescences per plant, and post-reproductive diameter were tested on the subset of plants receiving detailed morphological analysis with linear models using the lm function in R. For each of the linear-model analyses, we also tested models in which family was included as a nested effect within population, but family effects were not significant when models with and without the family component were compared using the anova function in R. Effects of population on the probability of forming rhizomatous shoots were tested in a 2×2 contingency table with Fisher’s exact test using the Fisher.test function.

Shoot-level variation in number of vegetative leaves and basal leaf length was also tested in mixed models using maximum likelihood, in which population was treated as a fixed effect, and plant was included as a random effect. Adding family as a random effect, with plant nested within family, did not significantly improve model fit. Mixed models were tested using the lmer function in the lme4 package in R. Significance of individual effects was evaluated by comparing models with and without the effect using the anova function in R.

We also tested the effects of branchiness rating, vegetative leaves per shoot, and bolting date on the number of inflorescences in linear models with and without population as an additional effect, using the lm function in R. Because the detailed shoot-level measurements were carried out over a three-week period, we also tested models in which the number of inflorescences was adjusted by the measurement date. For these tests, the number of inflorescences was first regressed on measurement date using lm, and the residual was then used as the dependent variable to test the effects of population, branchiness rating, vegetative leaves per shoot, or bolting date.

## Results

### Vegetative development and flowering

Mayodan plants produced visible inflorescences (bolted) and flowered much earlier than Spiterstulen plants (mean differences of 104 and 69 days, respectively; Table 2 and Fig. 2). Some Spiterstulen plants eventually bolted before daily photoperiods were increased from 9 to 16 h, but did not flower until after the switch to long days. Once the switch to long days was made, nearly all of the Spiterstulen plants began flowering over a short time span (days 273-277 after sowing). Mayodan plants also flowered earlier under field conditions in North Carolina, but by only one to two weeks [21]. In contrast to the growth chamber conditons, the North Carolina field site had daily average temperatures below 10 °C for approximately four months [33], possibly resulting in more complete vernalization, and had daily photoperiods of 11-13 h during the time period over which most bolting occurred.

**Table 2:** Vegetative development and reproductive timing in Mayodan vs. Spiterstulen plants

Trait	Mayodan		Spiterstulen		*P* value
	mean (± s.d.)		mean (± s.d.)		
	(*n* = 49)		(*n* = 19)		
Days to first bolting^a^	169.1	(±19.4)	273.2	(±1.0)	<0.0001^b^
Days to first flowering^a^	209.3	(±22.1)	278.3	(±1.0)	<0.0123^b^
Days to lateral vegetative development^a^	186.4	(±21.2)	252.7	(±7.7)	<0.0001^b^
Branchiness rating	1.57	(±0.67)	3.38	(±0.96)	<0.0001
Pre-reproductive rosette diameter (mm)	50.7	(±19.5)	135.9	(±33.9)	<0.0001^c^

^a^All days are relative to the date of sowing, with plants that did not show the trait excluded

^b^Wald test using Cox proportional hazard model, with plants that did not show the trait before dying or reaching the end of the experiment treated as censored data

^c^From linear model using log transformation

Visible lateral vegetative development, in the form of leaves that were clearly emerging from lateral shoots, also occurred earlier in Mayodan plants than in Spiterstulen plants by an average of 66 days (Table 2; Fig. 2). This was the opposite of the pattern we had predicted, based on patterns of vegetative diameter changes in North Carolina field data, which suggested stronger apical dominance in Mayodan plants [30]. However, the timing of lateral vegetative development relative to reproductive development was earlier in Spiterstulen plants than in Mayodan plants, consistent with the field study results. The mean date of visible lateral vegetative development was 20 days before the mean bolting date in Spiterstulen plants, but was 17 days after the mean bolting date in Mayodan plants (Table 2; Fig. 2). Visual ratings of vegetative branchiness at the time of bolting were also much higher in Spiterstulen than in Mayodan plants.

In contrast with the North Carolina field data [30], we found that pre-reproductive vegetative diameters were much larger in Spiterstulen than in Mayodan plants (Table 2). However, vegetative diameters were measured on the date of first bolting in the present study, which was much later on average in the Spiterstulen plants, while spring diameters had been measured over a short time period without regard to flowering status in the field study. A doubling of the bi-weekly fertilizer doses starting on day 207, during the interval between Mayodan and Spiterstulen bolting, produced a substantial growth response and counteracted any inverse relationships between rosette branching and rosette size.

### Development of lateral shoots

**Fig. 3:**
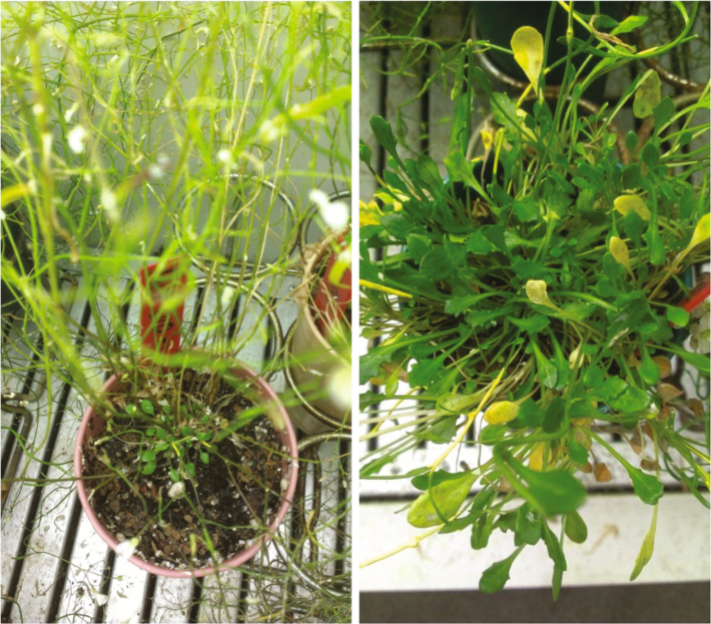
Comparison of vegetative rosettes late in the reproductive period on a typical plants from Mayodan (left) and Spiterstulen (right) populations

Mayodan and Spiterstulen plants showed contrasting visual patterns of vegetative development during their reproductive season, consistent with differences in perenniality. Senescence of older vegetative leaves on Mayodan plants as reproductive development proceeded was not compensated by development of new leaves, leaving vegetative crowns that consisted largely of dead foliage on most plants. By contrast, Spiterstulen plants maintained vigorous live vegetative crowns, even though dead leaves was visible on these plants too (Fig. 3). We analyzed the developmental patterns contributing to these striking visual differences between Spiterstulen and Mayodan plants by clipping lateral inflorescence-bearing shoots on a subset of plants from each population and characterizing the architecture of each shoot. In this subset of plants, post-reproductive vegetative diameters were significantly larger in Spiterstulen plants (Table 3). Post-reproductive vegetative crown size differences between populations reflected developmental differences during the reproductive period, because rosette leaves produced before reproduction had died by the time these measurements were taken.

**Table 3:** Lateral shoot development in Mayodan vs. Spiterstulen plants

Trait	Mayodan		Spiterstulen		*R* ^2^_pop_	*P* value
	mean (± s.d.)		mean (± s.d.)			
	(*n* = 27)		(*n* = 10)			
Post-reproductive rosette diameter (mm)	70.0	(±15.5)	144.3	(±19.3)	0.80	<0.0001
Plants with rhizomatous shoots/total plants	2/27	NA	8/10	NA	NA	<0.0001
Vegetative leaves per shoot	1.64	(±0.87)	10.55	(±2.52)	0.88	<0.0001
Longest leaf/shoot (mm)	21.9	(±8.2)	54.8	(±14.4)	0.69	<0.0001
Inflorescences per plant	30.4	(±8.8)	16.6	(±10.1)	0.32	0.0003

Plants from the two populations showed contrasting propensities to produce vegetative shoots from rhizomes. Eight of the 10 Spiterstulen plants analyzed had vegetative shoots emerging from rhizomes, with many rhizomatous shoots on some plants. By contrast, only two of the 27 Mayodan plants had any rhizomatous shoots (*P* = 0.00005 using Fisher’s exact test). Thus, production of rhizomatous shoots also appears to contribute to perenniality differences.

Higher-order shoots, and not just lateral shoots emerging from the primary rosette contributed to reproductive output and vegetative maintenance in both populations. Nearly all plants had inflorescences on both 2^nd^-order and 3^rd^-order shoots, with 4^th^ order inflorescences present on a few plants. The unelongated basal vegetative portions of many inflorescence-bearing shoots, which were produced prior to their reproductive transition, had additional higher-order vegetative shoots.

**Fig. 4:**
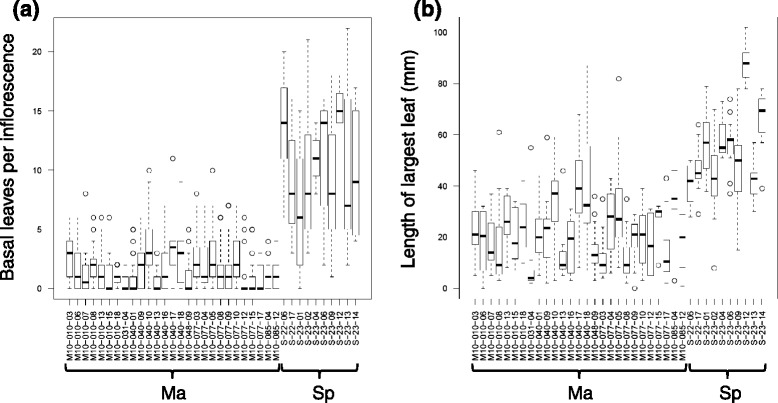
Distributions of **a** the mean number of basal vegetative-stage leaves per inflorescence, and **b** length of the largest basal leaf on each inflorescence, by plant

Inflorescences often formed from the apices of shoots that had previously been vegetative, especially on Spiterstulen plants, which was shown by the presence of well-developed leaves on the basal non-bolted portion of shoots. This was contrary to predictions of the strict meristem allocation hypothesis. However, the two populations showed large differences in the number of leaves produced on the basal vegetative portion of shoots before bolting, and in the size of these leaves (Fig. 4; Table 3). Shoots on Mayodan plants typically produced two or fewer small leaves before bolting, and in many cases the only evidence for vegetative leaves was the presence of higher-order shoots emerging from the vegetative portion of the shoot, implying one or more vegetative nodes. By contrast, shoots on Spiterstulen plants produced an average of approximately ten leaves before bolting, with the largest of these leaves averaging more than 50 mm long. Spiterstulen plants also had many well-developed lateral vegetative shoots in addition to the rhizomatous shoots, but these were not common on Mayodan plants.

**Fig. 5:**
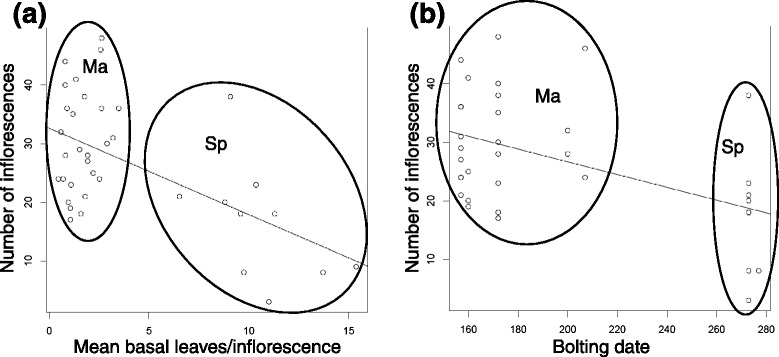
Scatterplots showing the regression of the number of inflorescences on **a** the mean number of basal leaves per inflorescence, and **b** the data of first bolting. Dotted ellipses show the phenotypic distributions of Spiterstulen (Sp) and Mayodan (Ma) plants

Mayodan plants produced nearly twice as many inflorescences on average than Spiterstulen plants (Table 3), similar to patterns observed in the field in North Carolina [21, 30], but the differences were less extreme in the current study. We tested whether variation in pre-reproductive apical dominance (branchiness) could explain these differences, as predicted under the hypothesis that early vegetative development quantitatively inhibits reproductive shoot development. Increases in branchiness showed a significant negative relationship with the number of inflorescences, but the effect was relatively weak (Table 4). Adding population as a factor significantly improved the fit of the model, indicating that branchiness was insufficient to explain the population differences in the number of inflorescences, contrary to predictions. By contrast, the mean number of basal leaves per shoot actually explained slightly more variance in the number of inflorescences than did population alone, and adding population as a factor in combination with the number of basal leaves did not significantly improve the model fit (Fig. 5a; Table 4). Later bolting also showed a significant negative effect on the number of inflorescences, and adding population to the model did not produce a significant improvement in model fit (Fig. 5b). However, bolting date explained substantially less variation in number of inflorescences than did population or the number of basal leaves (Table 4).

**Table 4:** Effects of developmental traits on number of inflorescences per plant

Independent variable	*R* ^2^	*P* value_pop_*
Population	0.320	NA
Branchiness rating	0.134	0.004
Vegetative leaves/shoot	0.330	0.58
Bolting date	0.236	0.13
Population (adj.)^a^	0.340	NA
Branchiness rating (adj.)^a^	0.168	0.004
Vegetative leaves/shoot (adj.)^a^	0.365	0.74
Bolting date (adj.)^a^	0.289	0.30

^a^Using the number of inflorescences per plant adjusted for measurement date

*P value for comparison of models with vs. without addition of population as a factor

The date on which the detailed inflorescence data were scored had a significant positive effect on the number of inflorescences (*P* = 0.01), suggesting that more inflorescences were emerging during the 21-day period in which data were collected on different plants. Consequently, we repeated the analyses above using the number of inflorescences adjusted for scoring date. Each of the traits above explained slightly higher proportions of variance in the adjusted number of inflorescences, but results were otherwise similar to those obtained without adjustment (Table 4).

## Discussion

### Mechanisms underlying life history variation

The most striking difference we found between *A. lyrata* populations was large differences in the number of basal leaves produced on lateral shoots before they undergo reproductive transition. In contrast with Spiterstulen plants, lateral shoots on Mayodan plants tended to become reproductive almost immediately, and basal leaves were usually tiny if present at all. These differences in turn were sufficient to explain the differences in reproductive output between populations. These results support previous modeling-based conclusions that resource allocation differences between *A. lyrata* populations are shaped by differences in vegetative development patterns [30]. However, we could reject the hypothesis that these developmental differences involved strict allocation of meristems to vegetative growth in lieu of reproduction. Lateral vegetative shoots were not precluded from bolting, as nearly all inflorescences on Spiterstulen plants had several to many well-developed leaves on their basal rosette portions produced prior to bolting. Higher-order lateral shoots were also common on plants from both populations, and showed developmental patterns similar to those of second-order shoots. This difference in the developmental timing of reproductive transition on individual shoots appeared to be largely responsible for the striking differences between the two populations in vegetative size and appearance during the reproductive phase (Fig. 3). These differences closely mirror the differences between populations in vegetative growth during the reproductive season observed in the previous field study [30]. The greater production of rhizomatous shoots on Spiterstulen plants probably also contributed to their larger post-reproductive vegetative diameter.

Our results were consistent with the hypothesis that early vegetative development on lateral shoots in Spiterstulen plants quantitatively inhibits inflorescence production, but not by the mechanism we had proposed. Pre-reproductive apical dominance failed to explain the population differences in number of inflorescences. Instead, the mean number of basal leaves per inflorescence was the best predictor for reduced numbers of inflorescences on individual plants, and even explained slightly more variation than did population. Thus, a delay in reproductive transition at the shoot level rather than earlier shoot initiation could be the primary factor reducing the number of inflorescences produced by Spiterstulen plants. The extended short-day conditions and the timing of changes in fertilizer dose probably counteracted genetic differences between populations in apical dominance, so we cannot rule out the possibility that apical dominance differences have additional effects on reproductive output.

The small sample size provided limited power to rule out other possible influences on inflorescence production. Bolting date was also sufficient to explain the population differences in number of inflorescences, but it explained less variation than did population alone or the mean number of basal leaves per inflorescence. The limited vernalization the plants received in the growth chamber may have contributed to the large differences between populations in bolting time, but photoperiod requirements were probably a larger factor. In previous studies, Spiterstulen plants showed relatively little response to vernalization compared to populations with more southerly origins in Europe [34].

The extended time of short photoperiods and low fertilizer doses, combined with the earlier flowering of the Mayodan plants, had the effect of decoupling resource acquisition and reproductive output. In contrast with field data from North Carolina [30], the earlier-flowering Mayodan plants had smaller vegetative crowns on average at the start of flowering than did Spiterstulen plants, but still produced more inflorescences. These results further support previous conclusions that acquiring more meristems or energetic reserves does not explain the greater reproductive output of Mayodan genotypes [30]. However, the expected tendency of larger plants to produce more meristems and thus more reproductive output [35, 36] might have been countered by the shorter time the later-flowering Spiterstulen plants had to produce inflorescences. QTL affecting resource allocation in the field were mostly separate from flowering time QTL [30], which suggests that bolting date, or its interactions with photoperiod and vernalization cues, is unlikely to be a major influence on the differences we observed in developmental patterns or reproductive output.

The large differences between populations in several traits (the number and size of basal leaves per inflorescence, production of rhizomatous shoots, and heterochrony between the start of vegetative vs. reproductive development) support previous conclusions that multiple genetic mechanisms contribute to divergent life history patterns [30]. Additional crosses and QTL analysis of a more detailed set of traits will be necessary to explain how each QTL region contributes to the developmental differences.

### Evolutionary implications of shoot development patterns

Patterns of resource allocation are usually interpreted in terms of limited morphological resources (e.g. meristems) or energetic resources that must be allocated between competing uses such as growth vs. reproduction [11, 13]. Our results in *A. lyrata*, combined with those of the earlier field studies [30], suggest an alternative model in which time may function as a limiting resource. Shoot reproductive transitions eventually taper off, probably in response to some combination of temperature extremes, photoperiod, or declining energy balances. After this point, new shoots that have not yet undergone reproductive transitions tend to remain vegetative. Genotypes that allocate a shorter period to vegetative development on shoots initiated during the reproductive season will have correspondingly more time for inflorescence development, and thus produce more inflorescences on average. We did not directly measure the time to reproductive transition on lateral shoots in this study. However, a positive relationship between the number of basal vegetative leaves per shoot and the time spend by shoots in the vegetative stage seems highly likely.

**Fig. 6:**
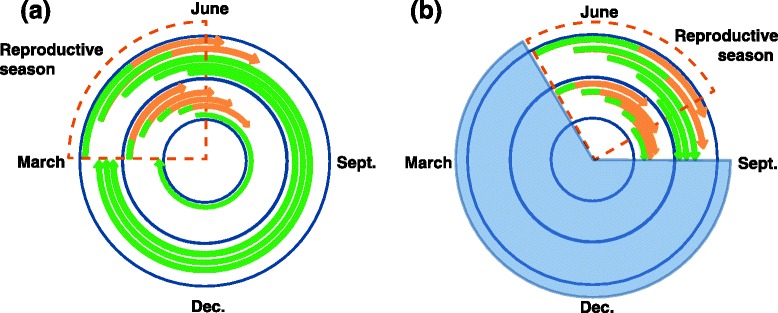
Proposed model for effects of contrasting genotypes on *A. lyrata* shoot development phenology under long and short growing seasons. Developing shoots are depicted by arrows in the diagrams, with the vegetative stage shown in green and the reproductive stage (inflorescence) in orange. Shoots undergo reproductive transitions only during the reproductive season bounded by temperature and photoperiod cues (orange dashed wedge). **a** Under long growing seasons, genotypes with short shoot vegetative stages (inner ring) will produce many inflorescences. A small subset of shoots initiating near the end of the reproductive season will not have time to acquire reproductive capability, and will thus remain vegetative afterwards. Subsequent vegetative development can occur on these shoots throughout the fall and possibly through the winter. Genotypes with long shoot vegetative stages (outer ring) will produce fewer inflorescences and have more shoots remaining vegetative at the end of the reproductive season. **b** Under short growing seasons, the reproductive season starts later in the spring, and temperatures are too cold for growth to occur during much of the year (blue shaded area). Genotypes with short shoot vegetative stages will thus have very limited opportunity for vegetative recovery after the reproductive season

The consequences of shoot development patterns for growth, reproduction and survival, and thus the trajectory of natural selection, will depend on the amount of time available for growth and reproduction in different climates. Projecting the shoot development patterns we found across the typical growing seasons in each parental environment provides insights into the possible basis for the fitness differences between populations seen in the field studies (Fig. 6).

In the model we propose, variation in shoot vegetative development changes the context of future developmental stages, analogous in some respects to processes that occur in the annual *A. thaliana* [37]. Genes directly affecting seed dormancy and germination in *A. thaliana* show cascading effects on flowering time and vice versa across generations, with environment-specific effects on reproductive output [37, 38]. Our results similarly suggest that alleles influencing the length of the shoot vegetative stage will affect the number of inflorescences the plant can produce in a finite reproductive season by shifting the seasonal timing of reproduction. In perennials, however, differences between plants in early development have additional effects on their adaptation to the post-flowering environment, potentially affecting survival and future reproduction. The genotypic differences we found in shoot development affect both the amount of leaf tissue and the number of vegetative meristems after flowering. In a warm environment, where the end of the reproductive period coincides with the start of summer and moisture-limited conditions, plants with shorter shoot development stages might have a survival advantage because they have less leaf tissue to exert transpirational demands. These plants will still have ample time in the late summer, fall, and possibly overwinter to produce new growth on the vegetative shoots they do have and rebuild energy reserves (Fig. 6a). By contrast, in cold environments the reproductive period ends in late summer or early fall when temperatures are already becoming colder. In these conditions, having little vegetative tissue at the end of flowering would be expected to reduce survival and future vigor, because there is little time for vegetative recovery before the return of cold temperatures (Fig. 6b).

This proposed model is consistent with the results of field studies [21, 30] and with the observed differences between Spiterstulen and Mayodan genotypes. In a single field season in North Carolina, all populations showed low post-reproductive survival regardless of vegetative size after flowering, so there may be little or no net fitness cost for a faster shoot reproductive transition. Consequently, natural selection is expected to favor evolution of shorter shoot vegetative stages in *A. lyrata* populations such as Mayodan from the warm extreme of its range. By contrast, Mayodan genotypes experienced high mortality when grown in Norway, and annual reproductive output of all genotypes was reduced in the colder Norway environment. These factors made survival a larger determinant of fitness in Norway, favoring selection for longer shoot vegetative stages such as seen in Spiterstulen genotypes. This model only takes into account genotypic differences in shoot vegetative development. Other factors such as photoperiod responses also probably differ between these populations [21, 23, 24]. Differences in critical photoperiod could shift both the beginning and the end of the reproductive season, in which case the bounds of the reproductive season shown in Fig. 6 would be different for the two populations.

The differences in developmental patterns we observed between the two populations show evidence of contributing to variation along a perenniality continuum. Some of the same Mayodan chromosomal segments in QTL regions that reduced vegetative development during the reproductive season in North Carolina were responsible for the reduced survival over multiple years in plants grown in Norway [29, 30]. We cannot be sure that the same genes were responsible for the effects observed in the two environments, but the results at least suggest that QTL for shoot development differences affect perenniality.

Moreover, the patterns of variation in shoot vegetative development we observed have a clear relationship to turnover-based concepts of perenniality, in which the quantitative balance between production vs. consumption of resources is a key life-history determinant [1, 3, 4]. In *A. lyrata* plants with a short shoot vegetative stage, new vegetative shoots are quickly consumed by reproductive transition. This reduces both the amount of photosynthetic tissue and the number of vegetative meristems persisting after flowering, which would place genotypes with short shoot vegetative stages closer to the annual extreme of the perenniality continuum. Reduced production of rhizomatous vegetative shoots in the Mayodan plants also contributes to the differences in perenniality patterns. *A. lyrata* from Mayodan are still perennial but clearly have a smaller margin of viability than do more reproductively-conservative genotypes. Thus, populations at the warm extreme of *A. lyrata*’s range might be in the process of evolving toward semelparity. Annual populations in *Erysimum capitatum* also occur in the warmest environments along an altitudinal gradient, though moisture differences may be more important than temperatures [7, 8]. In *Mimulus guttatus*, the opposite pattern is seen, in which annual populations are associated with shorter rather than longer growing seasons [5]. *M. guttatus* appears to have evolved a strategy of escaping cold conditions by reproducing quickly in alpine environments rather than maximizing its survival potential. Adaptive responses to environmental variation are likely to depend both on the exact nature of the environmental gradient and developmental characteristics of the ancestral population that influence evolutionary constraints [39].

## Conclusions

Our key finding here is that developmental differences between *A. lyrata* populations involve variation in the developmental timing of reproductive transitions on lateral shoots. These contrasting patterns potentially explain differences in reproductive output and perenniality between populations, and thus might make large contributions to adaptation to contrasting climates. Understanding life history variation in terms of time allocated to shoot vegetative development, rather than allocation of energetic resources or meristems, provides a potentially useful perspective for understanding perenniality.

Further research will be needed to identify the underlying genes and characterize their effects on fitness in contrasting environments, and also test the broader involvement of these life history differences among *A. lyrata* populations. Some of the resource allocation QTL regions identified by Remington et al. [30] also differentiate similar traits between Spiterstulen and more southerly European genotypes [34], suggesting broader relevance in adaptive evolution of *A. lyrata*. Genes regulating lateral shoot development have been shown to have key roles in evolution of plant architecture in maize domestication [40–42], and have been functionally implicated in life history differences between *Mimulus guttatus* populations [5]. Genes responsible for variation in lateral shoot architecture thus seem likely to function as “hotspot” genes with key roles in life history evolution [43, 44]. Consequently, identifying the responsible genes would provide novel insights on the degree of molecular parallelism in shoot architecture evolution, with broader implications for understanding the evolution of perenniality.

### Availability of supporting data

The data sets supporting the results of this article are available in the Dryad repository, http://dx.doi.org/10.5061/dryad.p732k.
